# Exploring Counselor Practices and Risk Assessment in a Proactive Digital Intervention Through Instagram in Young People: Qualitative Study

**DOI:** 10.2196/46579

**Published:** 2023-12-25

**Authors:** Natalie Peart, Sarah Hetrick, Kerry Gibson, Karolina Stasiak

**Affiliations:** 1 Department of Psychology University of Auckland Auckland New Zealand; 2 Department of Psychological Medicine University of Auckland Auckland New Zealand

**Keywords:** counseling, distress, empathize, internet, mental health, online text, proactive, qualitative study, risk assessment, self-harm, social media, suicide, validation, youth

## Abstract

**Background:**

Suicide is one of the leading causes of preventable death in young people, and the way young people are communicating suicidality has evolved to include web-based disclosures and help-seeking. To date, mental health intervention services, both on the web and in person, have been conceived in the traditional model, whereby support is provided if a young person (or their family) actively seeks out that support when distressed. On the other hand, proactive outreach is an innovative approach to intervention that has been shown to be effective in other areas of health care. Live for Tomorrow chat was delivered on Instagram and comprised of counselors who reach out to provide brief person-centered intervention to young people who post content indicating distress or suicidality.

**Objective:**

Our aim was to explore how counselors engaged young people in a proactive digital intervention and how risk assessment was conducted in this context.

**Methods:**

We analyzed 35 transcripts of conversations between counselors and young people aged 13-25 years using the 6-step approach of Braun and Clarke’s reflexive thematic analysis. These transcripts included a counseling intervention and a follow-up chat that was aimed at collecting feedback about the counseling intervention.

**Results:**

A total of 7 themes emerged: using microskills to facilitate conversations, building confidence and capacity to cope with change, seeking permission when approaching conversations about suicidality or self-harm, conversations about suicidality following a structured approach, providing assurances of confidentiality, validation of the experience of suicidality, and using conversations about suicidality to identify interventions. Counselors were able to translate counseling microskills and structured questioning regarding suicidality into a digital context. In particular, in the digital context, counselors would use the young person’s post and emojis to further conversations and build rapport.

**Conclusions:**

The findings highlight the importance of the counselor’s role to listen, empathize, validate, and empower young people and that all these skills can be transferred to a digital text counseling intervention. Counselors used a structured approach to understanding suicidality in a permission-seeking, validating, and confidential manner to identify interventions with the young person. These practices allowed the conversation to move beyond traditional risk assessment practices to meaningful conversations about suicidality. Moving beyond traditional risk assessment practices and into conversations about suicidality allowed for the validation of the young person’s experience and exploration of interventions and support that made sense and were seen to be helpful to the young person. This study highlighted the benefits of a proactive digital chat-based intervention, which is a novel approach to engaging with young people experiencing psychological distress and suicidality. Furthermore, this research demonstrates the feasibility and benefit of moving mental health intervention and support to a medium where young people are currently disclosing distress and intervening proactively.

## Introduction

### Prevalence of Suicidality in Young People

Suicide is a global phenomenon and was the fourth leading cause of death among those aged between 15 and 29 years in 2019 [1]. Approximately 222,093 young people aged between 10 and 29 years died by suicide in 2019. For each of these young people who died by suicide, it is estimated that 20 others may have attempted suicide [1]. Suicidal ideation is defined as the consideration of or desire to end one’s own life [2]. Suicidal ideation affects 14% of young people each year globally [3], and it is estimated to double the risk of suicide attempts [4]. Young people have higher levels of suicidal ideation than adults and, at the same time, lower levels of help-seeking behaviors [5]. The term suicidality encompasses the risk of suicide, usually indicated by suicidal ideation or intent, and will be used for this study due to its inclusivity of different experiences of suicide [6].

### Mental Health Service Use in Young People

There are many face-to-face services available for young people; however, young people report several barriers to service use, including shame, previous negative experiences, concerns regarding confidentiality from parents, a lack of control and agency, and the inflexibility of services [7,8].

In turn, the literature demonstrates that young people feel more comfortable disclosing their distress on the web [9], with those experiencing higher levels of suicidal ideation preferring web-based help-seeking [10]. Digital health interventions are becoming more common, especially in the post–COVID-19 era, with young people reporting how they value the anonymity and control offered in these interventions [7,11], in which young people perceive less judgment or authority from professionals and parents alike [9].

### Digital Interventions and Counseling

Digital mental health interventions are delivered through a range of digital platforms, including apps, websites, virtual reality, and live chat, and have been demonstrated to be more effective in retaining young people in mental health interventions than face-to-face outpatient care [12]. Digital interventions may also offer access to young people who otherwise may not seek help at in-person services, such as those who do not have financial, geographical, or familial support to access in-person services [13].

Core elements of counseling, such as therapeutic alliance [14] and counseling microskills [15], are essential to building rapport and trust with a young person, which have been established as essential in engaging young people in intervention on digital platforms [16]. Counseling microskills can be defined as key skills that develop rapport and assess and address the difficulty identified using skills such as active listening, open-ended questions, positive regard, and summarizing [15]. Research has also reported that many counseling microskills are adopted and easily implemented on the web [17,18] and that therapeutic alliance is equal to, if not higher, for digital interventions compared to face-to-face [14,19]. However, despite literature demonstrating otherwise, some clinicians doubt the efficacy of digital interventions, and in particular text counseling, compared to face-to-face interactions [20], highlighting a need for more research demonstrating how text counseling can use counselors’ existing skillset effectively.

### Suicide Risk Assessment and Digital Interventions

Traditionally, risk assessment involves asking a person about suicidal thoughts, their frequency and intensity, and the intent, plan, and access to means to act on these thoughts [21]. Risk assessment to classify levels of suicide risk has been demonstrated to have no discernible ability to predict the future risk of engaging in suicidal behavior [22]. However, services continue to use risk assessment to determine treatment access, which is not only invalidating for service users [23] but also allows clinicians to fixate on a classification of severity instead of engaging in compassionate, person-centered conversation.

A major area of clinician hesitancy to engage in digital interventions involves suicide risk and how to manage crises in a digital context. In particular, clinicians report feeling untrained in crisis management on the web [24] or that it was inappropriate to engage digitally with suicidal clients [11]. COVID-19 has resulted in an uptake of clinicians using telepsychology during the pandemic [25], and it is unclear whether this has resulted in a change of opinion about risk assessment and crisis management on the web.

### A Proactive Digital Intervention

To date, mental health intervention services both digitally and in person have been conceived in the traditional model, whereby support is provided if a young person (or their family) actively seeks out that support when distressed. Proactive outreach is an innovative approach to intervention that has been shown to be effective in other areas of health care, such as hard-to-reach injecting drug users, where HIV risk is reduced through street outreach programs providing needle disinfection and HIV testing [26]. Indigenous health services also use proactive responses, where cardiac health practitioners go into communities, build relationships, and administer portable interventions at people’s homes in rural communities [27]. Proactive approaches are already being used with an adolescent population and show high levels of engagement, such as a smoking cessation program through telephone outreach and helpline support, which reported a 61.5% to 80.5% engagement rate [28]. Proactive outreach provides intervention into communities where a need exists, reducing inequities [29], barriers [30], and the overall economic costs of the disease [31].

A proactive intervention approach with those experiencing suicidal ideation on the web has yet to be examined, and this research is the first to explore counselors’ practice and risk assessment in this context.

## Methods

### Aim and Research Questions

The aim of this qualitative study was to explore how counselors engaged young people in a proactive digital intervention and how risk assessment was conducted in this context.

### Study Setting and Data

Live For Tomorrow (LFT) is a nonprofit organization based in Aotearoa, New Zealand. LFT chat was a constituent of their work and was the “world’s first proactive helpline supporting teens in crisis on social media” [32]. LFT chat was operated through the social media platform Instagram, using an algorithm to search hashtags that indicated distress (ie, #depressed, #kms, and #suicidal). The algorithm identified posts that counselors used to contact the user who had posted, offering to listen and provide a proactive, person-centered brief intervention. Counselors were both volunteers and paid staff trained in counseling microskills, crisis management skills, and brief intervention.

In this study, we used transcripts from the digital chat that formed part of the counseling intervention, which also included a follow-up chat that was aimed at collecting feedback about the counseling intervention. These transcripts were part of routinely collected data about the service. The transcripts were collected between March 1, 2019, and February 29, 2020, based on inclusion criteria supplied to LFT. LFT then identified transcripts that met these criteria, anonymized them, and provided them to the researcher. The inclusion criteria were as follows: (1) the conversation involved a user aged between 13 and 25 years, (2) the counseling transcript contained a form of risk assessment, and (3) the young person was part of both a counseling conversation and a feedback conversation. The transcripts varied in length, with the final data set containing 274 pages and 79,155 words [33]. Detailed demographic information about the young persons was not collected; however, their age was collected, with a mean age of 16.7 years.

### Data Analysis

Reflexive thematic analysis using the 6-step approach of Braun and Clarke [34] was used to identify and report patterns within the data. The analysis involved an initial data familiarization phase followed by systematic data coding using NVivo 12 Pro (QSR International) to track iterations of the codes and quotes to remain true to the young person’s experience. This was followed by theme generation and developing, reviewing, and refining the themes before writing the results [34]. Although listed chronologically, these phases were moved back and forth between phases of the analysis. The thematic analysis took a social constructionist approach, acknowledging the perspectives of the researchers and the young people and accepting that distress and suicidal ideation may take on different meanings in different populations or cultural groups and at different times across a person’s lifetime. This paper’s analysis focused primarily on the counselor’s responses. Data analysis was led by the first author and assisted by the team, including a young person, through a process of consensus [35]. The analysis included recognized strategies for assuring the quality of qualitative research, including reflexivity [36]. This involved discussions between authors around different interpretations and acknowledgment of the first author: (1) being of New Zealand European ethnicity, (2) having had her own mental health journey including the use of helpline services, and (3) having previously worked as a counselor and supervisor for LFT.

### User Statistics

LFT provided user statistics to understand how the intervention was operating and, therefore, the context in which the live chats that were documented in the transcripts were conducted. These statistics were for the 1-year period within which the live chats documented in the transcripts were undertaken (Textbox 1).

**Service user data from March 1, 2019, to February 29, 2020.**

**Service user data (March 1, 2019 to February 29, 2020)**

- The number of posts reached out to was 4457.
- The average time between a young person’s post and a reach-out message from counselors was 10 minutes and 48 seconds.
- The number of young people who responded was 2105 (47.2%) out of 4457.
- The average time between a young person’s post and a reach-out message from counselors is 10 minutes and 48 seconds.

The lower number of transcripts used for this study compared to the overall number of young people that engaged in a conversation is due to the third inclusion criterion. This criterion required the young person to have engaged in a second follow-up feedback conversation with a counselor. Feedback was sought from young people who had engaged in a conversation within the previous 6 months, and we relied on the young person responding and providing feedback. Therefore, transcripts with young people who engaged in this feedback conversation were limited.

### Ethical Considerations

This study was approved by the Auckland Health Research Ethics Committee (AHREC; reference AH2840). Informed consent was sought from LFT for access to their routinely collected standard audit data and their consent to be named in this research. To protect the identity of the users, conversations were anonymized and deidentified by LFT staff before being provided to the researchers. LFT were compensated by the researchers for their staff’s time to anonymize and deidentify the transcripts.

## Results

### Overview

An overview of themes and subthemes is provided in Table 1.

**Table 1:** Overview of themes and subthemes.

Themes	Subthemes
Using microskills to facilitate conversation	Invitation to conversations Validation Reflection Open questions Summarizing Unconditional positive regard Emoji use
Building confidence and capacity to cope with change	Reinforcing existing strategies Provide encouragement Psychoeducation Provide strategies
Seeking permission when approaching conversations about suicide	Asking permission to talk about suicidal ideation or self-harm
Conversations about suicidality follow a structured approach	Ask about suicidal ideation Use of posts or tags to further conversation Ask about suicidal intent Ask about suicide plans Ask about access to means Safety planning
Providing assurances of confidentiality	Anonymity guaranteed due to nature of Instagram Confidentiality encouraged disclosure Fear of disclosing in real life
Validation of the experience of suicide	Counselors provide empathy Reflection of emotional distress Radical genuineness
Using conversations about suicidality to identify intervention	Use of scale rating questions Identify the young person's strengths Using conversation to model therapy interactions Using conversation to encourage further help-seeking

### Using Microskills to Facilitate Conversation

Counselors used counseling microskills to invite the young person into a conversation as part of their proactive reach out: “Hey, I was wondering if you wanted to chat? looks like things are tough for you atm...
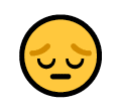
” (counselor 5) as well as inviting disclosure, “can I ask a bit more about why your parents don’t want you to talk to someone?” (counselor 6). Counseling microskills were used to provide opportunities for the young person to disclose their difficulties and explore their experience, including validation of the young person’s experience, “
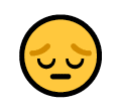
 There’s only so long you can bottle all those emotions up huh” (counselor 7), which led to subsequent disclosures:

that sounds like people really aren't giving you a break though...can’t imagine that makes it easier to deal with all the stress of high school as it is

Counselor 7

It doesn’t, like I really like helping people with their own problems but it gets so stressful with All my high school stuff , I’ve lost sleep and appetite over it.

Young person 17

Counselors often paired validation with reflective statements to extend a young person’s understanding of their experience: “
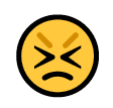
 that sounds really really frustrating friend. And a little bit hurtful that they don't take your feelings seriously?” (counselor 7). Reflection helped the young person explore their experience, rather than the counselor attempting to solve the problem right away.

it can be so hard to talk about something as difficult as suicide with people who don't really understand what you're going through...sometimes it can make you feel pretty alone, is that how it's been for you?

Counselor 22

Definitely, like my friends try to help but it always seems to make it worse

Young person 44

Counselors used open questions to create space and remain open to whatever the young person wanted to discuss. This was often seen at the start of the conversation: “Would you like to talk a bit about what’s going on with you at the moment?” (counselor 19); during risk assessment: “
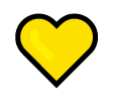
 What do you think it looks like, to not take it anymore?” (counselor 7); and when exploring interventions: “What do you think would be the best thing to happen now?” (counselor 7).

Summarizing was used less frequently by the counselors than the above techniques but was noted after the disclosure of a lot of information.

It sounds like moving house has caused a lot of distress for you :/ I’m also hearing that you’re feeling really unsure what the future looks like for you 
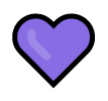
 Your Dad sounds incredibly difficult to be around, friend :(

Counselor 8

Summarizing was also used for clarification.

I just want to make sure I’ve got it clear - the two main sources of stress and pressure right now are school stuff and your friends using you as someone to tell their problems to, is that right?

Counselor 7

The counselors also used summarizing to create space for acknowledgment of more positive traits or experiences.

You are very resilient to have handled it on your own all this time, and it is wonderful that you have best friends in your life that are able to help you through some of the toughest moments

Counselor 32

Counselors used unconditional positive regard to create an environment that allowed the young person to feel safe to disclose, especially when discussing suicide and self-harm. “This is a judgement-free zone friend, that’s cool to hear you aren’t cutting 
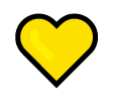
” (counselor 7). Unconditional positive regard allowed the young person to disclose further:

I love the emotional pain the torture...and i love the physical pain the cutting etc and most importantly the taste of blood...Sometimes I have to put in serious effort to stop myself from doing something Like once I wanted to cut my stomach open and eat my organs

Young person 55

Sounds pretty insane am I right?

Young person 55

Aw, not insane. I can imagine it would've been really hard to understand those feelings growing up!

Counselor 31

Emojis were used by counselors throughout conversations to convey meaning and emotion. Counselors often use emoji faces after hearing difficult stories from the young person: “all of those things you’re dealing with sound so exhausting 
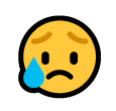
” (counselor 15), and to convey care: “Aw friend I can see you're in a really tough spot right now 
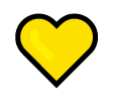
” (counselor 20). Emoji use appeared to replace nonverbal cues, for example, facial expressions and verbal cues such as “mmms,” in a digital space. Emojis were a unique addition to the counseling microskills used by the counselors.

### Building Confidence and Capacity to Cope With Change

Counselors encouraged self-efficacy by reinforcing the existing strategies that young people had already been using to cope with their distress.

All the great coping strategies you have…some connections and points of strength and support you can turn to when things feel like they're getting too much

Counselor 5

The counselors also provided encouragement to boost the young person’s confidence in themselves: “That's so cool, and you want to be a chef one day? You sound like a very creative person :)” (counselor 28). They also positively reinforced the young person’s gains: “that’s amazing that you have been clean for 4 weeks! I am really proud of you!” (counselor 6).

Psychoeducation contributed to the development of self-efficacy by providing the young person with the knowledge to understand their experience.

Well very generally depression keeps a cycle of thinking and behaving. It will be like negative thinking leads to a lack of motivation to act which can mean a lot of people tend to start doing less and less and this once again reinforces negative thinking

Counselor 7

Psychoeducation was used to create a sense of empathy for others.

Sometimes people who suffer from a head injury may end up acting in a different way and that can be soo difficult, especially when it is a loved one, like your sister...

Counselor8

It was also used to provide strategies that would assist the young person.

Write a list and make it realistic i.e for someone who cannot get out of bed day after day I would say for the first day maybe just have the task of getting to the letterbox and nothing else

Conselor 8

### Seeking Permission When Approaching Conversations About Suicidality or Self-Harm

The counselor would engage in conversation around suicidality or self-harm with a young person if their original post or digital chat indicated any level of suicidal ideation or engagement in self-harm. Many counselors started this conversation by asking permission and indicating exactly what they would be asking about: “If you don't mind me asking, do you ever think of suicide or self-harm?” (counselor 6). From the feedback given by the young person, the direct approach appeared to be valued: “at first i was hesitant like talking about my mental state to a stranger...but then I welcomed it” (young person 21).

These permission-seeking questions were always answered with disclosures by the young person:

I know this is a heavy question, but can I ask if suicide is something you've ever thought about? 
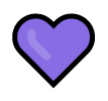

Counselor 4

I’ve attempted suicide twice My parents have no clue And my scars are visible I just don’t think they care bc I’m pretty sure they’ve seen them

Young person 9

Aww friend xx you must have been in such a dark place to have felt like suicide was the only way out :(

Counselor 4

### Conversations About Suicidality Follow a Structured Approach

Counselors used a structured approach to conversations about suicidality, following a structure often implemented in in-person services. Counselors initially asked about suicidal ideation: “sometimes when people experience these kinds of trauma they can get suicidal thoughts. Have you been experiencing any of these?” (counselor 20). Counselors were able to refer to the Instagram post if suicidal ideation or self-harm were not initially disclosed: “If you don't mind me asking, your post had a suicide tag, is that something you think about a bit?” (counselor 3). If the young person had suicidal ideation, suicidal intent was explored.

on a scale of 1 - 10 where 1 was you had no intention of attempting suicide today and 10 was you have strong intention to attempt suicide tonight, what number would you give yourself?

Counselor 22

Subsequent exploration of any plans was explored, as seen in the interaction below:

Do you have any kind of plan for how you may carry out these suicidal thoughts?

Counselor 20

*Yes* [Young person 40]

Do you mind telling me?

Counselor 20

I would just … until I pass out and hope I dont wake up

Young person 40

Lastly, the counselor would ask whether they had any access to means to carry out their plan and safety, “is there anyone at home atm?” (counselor 9) or “do you think you will be safe or will you be able to reach out if you felt you weren’t?” (counselor 8).

### Providing Assurances of Confidentiality

Due to the nature of Instagram, the counselors were unable to identify who the young person was or where they were located. This allowed for a high level of confidentiality because the counselor, even in situations where an in-person service would trigger a breach of confidentiality, could not disclose this information.

you mentioned you were suicidal - sounds like you're in such a dark place to be feeling that way :( do you mind telling me if that's something you're planning to carry out in the near future?

Counselor 22

U won't tell anyone?

Young person 44

there's not really anyone I can tell <3 I'm worried for your safety though

Counselor 22

I'm not sure, I've attempted it before but that failed

Young person 44

This high level of confidentiality increased the young person’s amount of disclosure: “talking to someone online is a bit easier since not to be rude but they don’t really know much about you and it’s easy to get things off your chest” (young person 17) and reduced the fear that young people had of disclosing content in real life: “If I told any of the professionals ab the stuff that goes on in my head they would put me in a mental hospital” (young person 55).

### Validation of the Experience of Suicidality

Where suicidal ideation or self-harm was disclosed, counselors demonstrated empathy and validated the experiences of what had led the young person to feel suicidal (without validating the act of suicide itself). “I think that wanting to ‘disappear’ is a really understandable response for wanting to leave behind the pain that you feel every day” (counselor 32). Counselors appeared to use several different levels of validation. Accurate reflections appeared in many risk assessments, with counselors validating the emotional distress that comes with suicidal thoughts: “That is a heavy conclusion to come to friend and I can imagine that would have taken quite a few strong thoughts to get there...” (counselor 22). Alongside this validation, counselors demonstrated radical genuineness in their validation of the young person being able to disclose their suicidality: “I'm really glad we could chat - I feel honoured that you've felt able to be so open and honest with me 
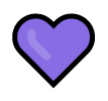
 you're such a brave human xx” (counselor 4).

### Using Conversations About Suicidality to Identify Interventions

In conversations about suicidality, the counselor used the opportunity to identify appropriate interventions in almost every transcript. Counselors would use a scale rating question to understand the intent of suicidal behavior and to talk about protective factors: “So with a scale of 6.5 I would guess there is something keeping you around?” (counselor 21), which subsequently enabled the encouragement of identifying the young person’s strengths to prevent suicide attempts:

I’m not talking to my mom bc I don’t want to worry her, but I’ll try to tell her

Young person 19

Hey that’s really cool you’re thinking about telling your Mum - from what you said earlier it does sound like she cares.

Counselor 8

Yes she cares a lot my siblings and I, she’s the best person I’ve ever met. My dad used to mistreat physically and emotionally my siblings and I but I love him too.

Young person 19

(Following day) Hello, I talked with my mom. She helped me a lot. You gave me the strength to tell her even though it was extremely hard for me

Young person 19

The relationship between the counselor and the young person was also used to model how therapeutic interactions might happen in other services, overcoming some of the barriers and misconceptions that young people had.

it can be so relieving when we are able to safely express how we're feeling and what's happened/happening in our lives, hey? Being listened to and heard can be so powerful xx how would you feel about talking to someone else on an online platform like this?

Counselor 4

This comparison allowed the young person to understand what a help-seeking experience could be like, increasing the young person’s confidence to use it in the future.

Okay so there are a couple of places you could try...[Local helpline information provided] I really hope you find the right support. You deserve to feel really good <3

Counselor 22

Yeah, but like I said yesterday I'm terrible at talking to people about stuff like this

Young person 44

You’ve done amazingly talking to us - do what you've done with us - be honest and open as much as you can.

Counselor 22

Yeah I guess that’s the only way

Young person 44

## Discussion

### Overview

This study aimed to explore how counselors engaged young people in a novel proactive digital chat intervention and how, what is traditionally referred to as risk assessment, was conducted in this context. Using a qualitative framework, a total of 7 key themes highlighted how counselors used counseling microskills to facilitate supportive conversations and encourage building the young person’s confidence and capacity for change. A change in approach from risk assessment with the intent of risk classification to conversations about suicidality allowed the counselors to continue to use the effective structured questioning that has typically been used in risk assessment practices [37], but with the ultimate aim and outcome of identifying interventions with the young person. These conversations around suicidality included first seeking permission, with the context on Instagram ensuring confidentiality, and validating the young person’s experience of suicidality.

As this was not a traditional help-seeking model, proactive outreach meant counselors needed to overcome the initial barrier that the young person may not be as receptive as those who present at a mental health service. This proactive outreach approach demonstrated a 47.2% (2105/4457) response rate and a 26% (1159/4457) engagement rate, which is the first data of its kind for a proactive digital counseling intervention. This is lower than other areas of proactive approach with adolescents [28]. However, these lower rates are consistent with the literature demonstrating that young people engage in mental health services at a lower rate than adults [38].

This approach also highlighted the benefit of being on the web in a space where young people are disclosing distress and suicidal ideation to peers. Proactive support was offered by counselors within an average of 10 minutes and 48 seconds of the creation of the post. This ability to respond to distress when it is occurring results in a highly accessible service, which young people have previously cited as a barrier in traditional mental health care [39]. By providing almost real-time and therefore early intervention with young people, this proactive counseling model demonstrates best practice [40] and potentially reduces the risk of further distress and the development of psychological disorders [41].

The proactive nature meant that the first interaction with the young person focused on creating a supportive conversation, allowing the person autonomy to engage and disclose as they wished. This invitation allowed for a transfer of power to the young person as they could decide on their level of engagement, contrary to power dynamics that often favor the adult or professional in face-to-face services [39]. The anonymity offered by Instagram resulted in an equalizing of power in the therapeutic relationship [42], as the young person was able to discuss topics that they may not ordinarily discuss [39,43].

Counselors used counseling microskills like those used in in-person services to construct therapeutic alliances; however, they required some adaptation to a digital chat environment. Both counselors and young people used emojis to express emotion and replace nonverbal cues [44]. Counselors were able to build trust through validation and reflection, which encouraged the young person’s ability to make change in their life and reinforced coping strategies. Using validation as the method of connection with the young person became the core of the relationship, which demonstrated care rather than giving advice [16].

Counselors used a structured approach to conversations about suicidality and self-harm behavior with the intention of focusing on intervention with the young person and not as a risk screening tool, in line with recent evidence [22]. Counselors had conversations about suicidality using a person-centered approach by listening, understanding, empathizing, and helping the young person find solutions they could connect with in a meaningful way. By having these conversations in a web-based, confidential environment, young people’s concerns about the consequences of disclosure in a face-to-face context can be minimized [45]. By moving away from risk assessment for the purpose of risk classification, the associated consequences of being classified in a certain way are removed, for example, being deemed low-risk and therefore not eligible for treatment [22,46]. LFT facilitated a real movement toward understanding the distress leading to suicidality and strategies to mitigate that distress instead of focusing on assessing the “risk” of future self-harm and suicide alone. This approach granted young people the opportunity to discuss and understand their suicidality, and together with the counselor, they focused on intervention strategies.

### Limitations

A key limitation of this study is that the data were extracted from transcripts and not interviews, which limited the exploration that could be conducted. The transcripts themselves ranged in length, from a brief conversation that would last 30 minutes to a continued conversation over many days. This analysis did not examine how the young person experienced this interaction and whether it was effective from their perspective. LFT managers gathered feedback on their service 6 months postintervention, which may have affected recall. Furthermore, as LFT staff themselves gathered feedback, this may have influenced the young person’s desire to provide honest feedback.

### Conclusions

This study provided important insight into how counselors engage young people, use counseling microskills, and move beyond traditional risk assessment to meaningful conversations about suicidality and intervention in an innovative, proactive approach to mental health intervention on Instagram. This study found that LFT counselors could use different microskills to facilitate supportive conversations and encourage self-efficacy in young people. Counselors used a structured approach to understanding suicidality in a permission-seeking, validating, and confidential manner to identify interventions with the young person. These practices allowed the young person to retain control and power in the conversation as well as demonstrate that a proactive, person-centered approach is possible within a digital chat-based environment. Services such as LFT have an important place in the range of services that must be available to young people in distress.

## Abbreviations

AHREC: Auckland Health Research Ethics Committee
LFT: Live For Tomorrow

## Data Availability

The data sets analyzed during the current study are available from the corresponding author on reasonable request.
